# Predictive factors for hospital self-discharge in tuberculosis admissions in the state of Rio de Janeiro, from 2011-2018: a retrospective cohort study

**DOI:** 10.1590/S2237-96222024v33e20231202.en

**Published:** 2024-10-14

**Authors:** Marcela Bhering, Caroline Millon, Maria Eduarda Beltrão da Rosa Rinaldi, Hedi Marinho de Melo Guedes de Oliveira

**Affiliations:** 1 Fundação Oswaldo Cruz, Escola Nacional de Saúde Pública Sergio Arouca, Rio de Janeiro, RJ, Brazil; 2 Universidade do Grande Rio, Curso de Medicina, Rio de Janeiro, RJ, Brazil

**Keywords:** Tuberculosis, Evasión del Paciente, Pacientes Internos, Factores de Riesgo, Vulnerabilidad Social, Adhesión al Tratamiento, Tuberculosis, Patient Dropouts, Inpatients, Risk Factors, Social Vulnerability, Treatment Adherence

## Abstract

**Objective:**

To assess factors associated with hospital self-discharge of patients with tuberculosis in the state of Rio de Janeiro, Brazil, 2011-2018.

**Methods:**

This was a retrospective cohort study in a referral hospital. Clinical and epidemiological characteristics were compared according to hospitalization outcome (self-discharge, formal discharge, or death). Hazard ratios (HR) with 95% confidence intervals (95%CI) for the association of self-discharge with explanatory variables were estimated using Cox regression.

**Results:**

Of the 1429 hospitalizations, 10.4% ended in self-discharge. Female sex (HR = 1.47; 95%CI 1.03;2.11), age ≤ 42 years (HR = 2.01; 95%CI 1.38; 2.93), substance use (HR = 1.62; 95%CI 1.12; 2.34), hospitalization after treatment dropout (HR = 2.04; 95%CI 1.37; 3.04), and homelessness (HR = 2.5; 95%CI 1.69; 3.69) were associated with self-discharge.

**Conclusion:**

Patients with social vulnerability require more careful monitoring during hospitalization.

## Study contributions

**Main results:**

Homeless people, illicit drug use, female sex and history of dropout from previous treatment showed association with hospital self-discharge in patients with tuberculosis admitted to a reference hospital in the state of Rio de Janeiro.

**Implications for services:**

Need for more comprehensive support for vulnerable patients, in addition to promoting treatment adherence and training health professionals to deal with the complex psychosocial issues related to tuberculosis.

**Perspectives:**

It is crucial to develop public policies that consider social factors in tuberculosis management, as well as promoting cooperation and multisectoral approaches to address both tuberculosis and underlying social issues.

## RESUMEN

**Objetivo:**

Analizar los factores asociados con la deserción hospitalaria de pacientes con tuberculosis en el estado de Río de Janeiro, en 2011 y 2018.

**Métodos:**

Estudio de cohorte retrospectivo en un hospital de referencia. Se compararon las características clínicas y epidemiológicas según el desenlace (deserción, alta o fallecimiento). Se estimaron los hazard ratios (HR) con intervalos de confianza del 95% (IC_95_%) para la asociación de la deserción con las variables explicativas mediante regresión de Cox.

**Resultados:**

De las 1429 hospitalizaciones, 10,4% desertaron. El sexo femenino (HR= 1,47; IC_95_% 1,03; 2,11), la edad ≤ 42 años (HR = 2,01; IC_95_% 1,38; 2,93), el consumo de drogas (HR=1,62; IC_95_% 1,12;2,34), la hospitalización después del abandono del tratamiento (HR = 2,04; IC_95_% 1,37; 3,04) y la situación de calle (HR = 2,5; IC_95_% 1,69; 3,69) se asociaron con la deserción.

**Conclusión:**

Pacientes vulnerables requieren un monitoreo más cuidadoso durante la hospitalización.

## INTRODUCTION

Tuberculosis continues to be a serious public health problem. The World Health Organization (WHO) estimated that in 2022, 10.6 million people became ill worldwide and around 1.3 million people died from tuberculosis, including 187,000 among people living with HIV.^1^ Brazil is considered a priority country regarding tuberculosis control due to the high burden of the disease and HIV co-infection.^1^ Given the need to reduce the burden of this disease, the WHO has highlighted the importance of combating tuberculosis among vulnerable populations and the need for effective interventions at national and global level.^1^

In Brazil, diagnosis and treatment are offered free of charge and the Ministry of Health recommends that new and retreatment cases using a basic regimen, cases of relapse after cure and re-entry after therapeutic interruption, be treated on an outpatient basis in the primary health care network. Cases that require special therapeutic regimens, due to toxicity, intolerance or contraindications to the adoption of the basic regimen, and assessment of therapeutic failure, should be treated in secondary care referral units, while cases of proven resistance or therapeutic failure due to resistance should be referred to tertiary care units.^2^

Despite not playing a central role in the treatment of tuberculosis, hospitals are still an important gateway for diagnosing the disease, especially in medium and large urban centers.^2,3^ The proportion of tuberculosis cases diagnosed at the hospital level is higher than expected in several regions of the country, either as a result of barriers to accessing primary health care or due to delays in diagnosis.^4^ It is estimated that 30% of tuberculosis cases are diagnosed in hospitals, after worsening and requiring hospitalization, resulting in higher medical expenses and increased mortality rates.^4,5^

It is well known that treatment interruption is an enormous challenge to achieving the goals recommended by the WHO in its plan to end tuberculosis.^1,6^ According to studies conducted, the factors associated with interrupting tuberculosis treatment are multiple and, therefore, in general, they are associated with the patient (HIV co-infection, use of alcohol, tobacco and drugs), socioeconomic factors (low education, homelessness), the type of treatment used and its implementation in health services (failure to use directly observed therapy).^7^

In Brazil, the Ministry of Health recommends hospitalization for the treatment of tuberculosis in cases where there is hemoptysis, deterioration of the patient’s general condition, presence of significant comorbidities, severe intolerance to medications, development of drug-induced hepatitis and situations of social vulnerability.^8^ In long-stay hospitals, patients often need to stay for the entire period of treatment and/or to restore their health, due to precarious housing conditions, lack of family support or other difficulties related to the social context.^8^ In the hospital context, self-discharge in cases of hospitalizations due to tuberculosis, defined as the patient leaving the hospital environment without medical authorization and without informing the admissions department,^9^ becomes an important cause of treatment interruption, contributing to unfavorable outcomes, such as multidrug resistance and death.^4^

Self-discharge is a challenge for disease control and a relevant problem in the management of public health strategies, since interruption of the appropriate course of treatment can lead to a worsening of the patient’s health status, increased risk of readmissions and even death. Furthermore, it can result in greater community transmission of the disease, compromise the effectiveness of medications, and increase the risk of drug resistance. Although there are studies in the literature on dropout from tuberculosis treatment,^7^ there are no studies that investigate self-discharge of patients hospitalized due to tuberculosis. Therefore, this study aims to identify factors associated with hospital self-discharge among patients with tuberculosis. 

## METHODS

This is a retrospective cohort study carried out at *Hospital Estadual Santa Maria* with data from patients admitted between January 2011 and December 2018. 

The hospital has 77 beds dedicated exclusively to the hospitalization of adult patients with tuberculosis, HIV co-infection, multidrug-resistant tuberculosis (MDR-TB), with an average of 200 hospitalizations per year. As it is a referral hospital, patients are referred there when diagnosed as having active tuberculosis. Of the two tuberculosis referral hospital units in the state of Rio de Janeiro, it is the only one located in the city of Rio de Janeiro (state capital), with an average of 200 hospitalizations per year during the study period. The state of Rio de Janeiro has high incidence of tuberculosis cases, with a rate of 70.7 cases per 100,000 inhabitants.^10^

The data were collected directly from medical records by two female public health physicians from the hospital’s Epidemiology Center, responsible for epidemiological surveillance of cases.

The reference population consisted of adult patients (≥ 18 years of age) hospitalized during the study period. All patients with confirmed diagnosis of tuberculosis were included, either by detection by direct test (Ziehl-Neelsen method), a positive culture result for *Mycobacterium tuberculosis*, or by the presence of clinical, epidemiological and radiographic findings compatible with tuberculosis, according to the location of the disease.

The outcomes were classified according to the type of discharge from hospital, in accordance with Ordinance No. 312, of April 30, 2002:^9^ self-discharge – patient leaving hospital without medical authorization and without informing the sector in which they were hospitalized; formal discharge – medical act that determines the end of hospital stay, which can be either when the patient is cured or has improved; death – occurring after admission to hospital, having tuberculosis as one of the causes of death. 

The following explanatory variables were selected from the medical records and included in the study, following a previously published rationale:^11^

Sociodemographic variables
sex (male; female);
age group (in years: 18-30; 31-44; 45-64; ≥ 65);
race/skin color (White, Black, mixed race, Asian, Indigenous, unknown);
schooling (in years: none; 1-3; 4-7; 811; ≥ 12);
resides in the state capital (yes; no).

Clinical variables
clinical form of tuberculosis (pulmonary; extrapulmonary; both);
multidrug-resistant tuberculosis (resistance to at least rifampicin and isoniazid) (yes; no);
type of admission (new case; post-dropout; transfer).

Comorbidity variables
HIV (positive, negative, test not performed);
diabetes (yes; no);

Risk factor variables
alcohol dependence (yes; no);
illicit drug use (yes; no);
tobacco smoking (yes; no);
return to treatment after dropout (yes; no);
street dweller or coming from a public shelter (yes; no).

Binary variables were used for age (≤ 42 years) and schooling (< 8 ≥ 8 years) for performing the regression analysis, defined based on previous studies and tested for the best fit of the model.^12,13^ In the case of variables for which more than 10% of the data was incomplete (schooling, resident in the state capital, form of tuberculosis), multiple imputation was performed by regression using the predicted mean matching method to predict missing data.^14^

Given the nature of the variables, absolute and relative frequencies were used to describe the characteristics of the patients and the occurrence or not of the outcome. Fisher’s exact test was used to compare the proportions between the group that self-discharged and the groups that were formally discharged or died. The medians and interquartile range (25-75) of length of stay were also calculated according to the type of hospital discharge.

Taking the same set of participants, Cox proportional hazards models were used to estimate the hazard ratio (HR) between the self-discharge outcome and the explanatory variables. The cohort time zero was defined by the date of hospitalization and the follow-up time corresponded to the period from the date of hospitalization until the outcome. In the case of patients who did not have the outcome of interest (self-discharge), data were censored at the last known follow-up date.

The Hosmer and Lemeshow purposive selection method was applied, which is similar to the backward selection method.^15^ In the initial model, variables with p-value ≤ 0.20 were included in the univariate analysis, which allows a comprehensive initial exploration of associations with the dependent variable. This helps identify potential factors of interest, minimizing the risk of type II error and prioritizing variables for further analysis along with theory and variable refinement supported by literature. In the next step, the variables for which the null hypothesis was not rejected and which did not cause a change greater than 20% in the coefficient estimates were removed from the model, one at a time. In the adjusted model, variables associated with the outcome with significance ≤ 0.10 were included. The model with the best data fit was chosen using the likelihood ratio test. Schoenfeld residuals were used to test the proportional hazard assumption and Cox-Snell residuals were used to assess the model’s overall goodness of fit. A fixed significance level of 5% was taken as the cutoff point for all statistical tests. To evaluate the discriminative capacity of the model, Harrell’s C statistic and the ROC (receiver operating characteristic) curve were used. Statistical analyses were performed using Stata version 13.1.

The study was approved by the Research Ethics Committee of the *Escola Nacional de Saúde Pública Sergio Arouca/Fiocruz* (Certificate of Submission for Ethical Appraisal No. 44735121.5.0000.5240), as per Opinion No. 4.641.397 dated Abril 9, 2021.

## RESULTS

**Figure 1:**
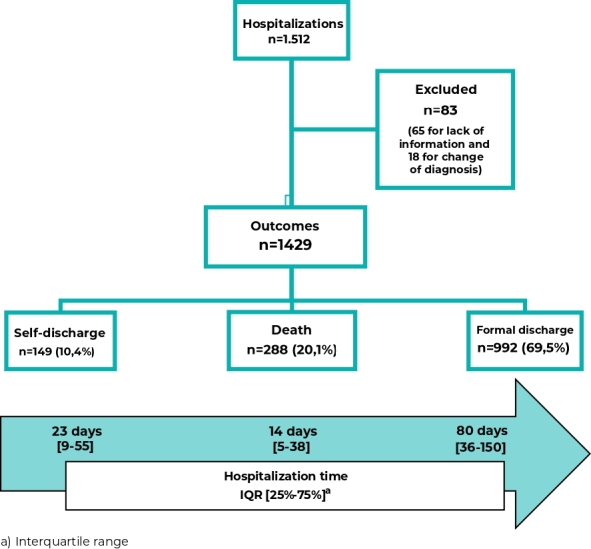
Patient frequency, median time and interquartile range (in days), according to outcome, Rio de Janeiro, Brazil, 2011-2018 (n= 1,429)

Between 2011 and 2018, 1,512 patients were admitted to the hospital. Sixty-five records were excluded due to lack of complete information and a further 18 that were not cases of tuberculosis (change of diagnosis), leaving 1,429 records. Of the total 1,429 cases, 149 (10.4%) were self-discharged, 288 (20.1%) died and 992 (69.5%) were discharged formally, 145 (10.2%) due to cure and 847 (59.3%) due to clinical improvement. Overall, the median length of stay was 59 days (interquartile range 19-121) and 23 days for self-discharge (Figure 1). 

The city of Rio de Janeiro accounted for 72.2% of the cases analyzed, followed by the *Baixada Fluminense* region with 226 (19.3%). Duque de Caxias and São João de Meriti were the *Baixada Fluminense* municipalities that had the most hospitalized patients, 66 (5.6%) and 56 (4.7%), respectively.

Table 1 describes the sociodemographic and clinical characteristics of the study population by outcome (self-discharge, death or formal discharge). Overall, 1,063 (74%) patients were male. Female patients were proportionally more present among the cases of self-discharge (30.9%), than in the formal discharge (25%) and death (21.9%) groups, but without a statistically significant difference (p = 0.115). Among the hospitalizations analyzed, median age was 42 years (interquartile range 32-54) among patients in general and 35 years (interquartile range 30-43) among those who self-discharged. 

**Table 1:** Participant sociodemographic and clinical characteristics per type of hospital discharge, Rio de Janeiro, Brazil, 2011-2018 (n=1,429)

Characteristics	Self-discharge (%) N = 149	Death (%) N = 288	Formal discharge (%) N = 992	p-value^a^
**Sex**				
Male	103 (69.1)	225 (78.1)	735 (75)	0.115
Female	46 (30.9)	63 (21.9)	257 (25)	
**Age group**				
18-30	37 (25.5)	36 (12.7)	213 (21.9)	< 0.001
31-44	73 (50.3)	68 (24.0)	338 (34.8)	
45-64	34 (23.5)	136 (48.1)	372 (38.3)	
= 65	1 (0.7)	43 (15.2)	48 (5.0)	
**Race/skin color**				
White	24 (18.2)	71 (26.1)	201 (21.9)	0.335
Mixed race	56 (42.4)	112 (41.2)	407 (44.4)	
Black	52 (39.4)	89 (32.7)	308 (33.6)	
**Schooling (years)**				
None	7 (12.7)	13 (9.7)	35 (8.6)	0.665
1-3	19 (34.6)	43 (32.1)	140 (34.2)	
4-7	15 (27.3)	48 (35.8)	120 (29.3)	
8-11	11 (20)	18 (13.4)	67 (16.4)	
= 12	3 (5.4)	12 (9.0)	35 (8.6)	
**Resident in the state capital**				
Yes	91 (79.8)	171 (68.7)	586 (73.6)	0.074
No	23 (20.2)	78 (32.3)	210 (26.4)	
**Street/shelter dweller**				
Yes	36 (24.2)	13 (4.5)	83 (8.4)	< 0.001
No	113 (75.8)	275 (95.5)	909 (91.6)	
**Clinical form of tuberculosis**				
Pulmonary	113 (99.1)	224 (96.1)	707 (95.4)	0.581
Extrapulmonary	0 (0.0)	3 (1.3)	12 (1.6)	
Both	1 (0.9)	6 (2.6)	22 (3.0)	
**Other factors**				
MDR-TB	34 (22.8)	52 (18.1)	193 (19.5)	0.481
HIV positive	37 (24.8)	79 (27.4)	241 (24.3)	0.549
Diabetes	8 (5.3)	31 (10.7)	123 (12.4)	0.030
Alcohol abuse	86 (57.7)	149 (51.7)	557 (56.2)	0.352
Ilicit drug use	97 (65.1)	74 (25.7)	437 (44.1)	< 0.001
Tobacco smoking	90 (60.4)	142 (49.3)	583 (58.8)	0.012
**Type of admission** ^b^				
New case	18 (13.6)	55 (21.2)	196 (21.4)	<0.001
Post-dropout	34 (25.8)	25 (9.7)	106 (11.2)	
Transfer	80 (60.6)	179 (69.1)	614 (67.0)	

a) Fisher’s exact test; b) Variable with less information due to medical record data incompleteness.

Among cases whose level of schooling was known (n = 598), 440 (73.6%) had up to seven years of schooling. Among dropout cases there was a higher proportion with no schooling (12.7%), than among cases that died (9.7%) and were discharged formally (8.6%), with no significant difference between the groups 

(p = 0.665).

Among the self-discharge cases, 24.2% were people living on the streets or had come from shelters; for this population formal discharge and death were lower, 8.4% and 4.5%, respectively (p < 0.001). Patients who used illicit drugs were proportionally more likely to drop out (65.4%) than to be discharged (44.1%) and die (25.7%; p < 0.001). The same occurred among patients who were smokers, with a higher proportion of cases of self-discharge (60.4%), than of formal discharge (58.8%) and death (49.3%; p = 0.012). 

There was a higher percentage of MDR-TB cases in the self-discharge outcome (22.8%). In the death outcome, there was a higher proportion of HIV positive cases (27.4%), while in formal discharges there were more diabetes cases (12.4%). The frequency of patients who had dropped out of previous treatment was higher among self-discharge cases (25.8%), when compared to cases of death (9.7%) and formal discharge (11.2%) (p < 0.001). 

After adjustments, female individuals had higher risk of self-discharge (HR = 1.47; 95% CI 1.03;2.11) when compared to male individuals (Table 2). Patients aged ≤ 42 years had twice the risk of self-discharge compared to older patients (HR = 2.01; 95%CI 1.38;2.93). People living on the streets or in shelters were more at risk of self-discharge (HR = 2.50; 95%CI 1.69;3.69) than patients with a known place of residence. 

**Table 2:** Predictive factors of self-discharge among inpatients at a tuberculosis referral hospital, Rio de Janeiro, Brazil, 2011-2018 (n=1,429)

Variable	Crude measure of association (95%CI) HRc^a^ 95%CI			p-value	Adjusted measure of association (95%CI) HRa^b^ 95%CI	p-value
**Sex**						
Male	1.00				1.00	
Female	1.54 (1.08;2.19)		0.016		1.47 (1.03;2.11)	0.033
**Age = 42 years**						
No	1.00				1.00	
Yes	2.54 (1.78;3.62)		< 0.001		2.01 (1.38;2.93)	< 0.001
**Race/skin color**						
White	1.00					
Mixed race/Black	1.22 (0.77;1.92)		0.379			
**Schooling (years)**						
< 8	1.00					
= 8	0.91 (0.49;1.68)	0.779				
**Resident in the state capital**						
No	1.00					
Yes	1.39 (0.88;2.21)			0.152		
**Street or shelter dweller**						
No	1.00				1.00	
Yes	2.99 (2.05;4.36)			< 0.001	2.50 (1.69;3.69)	< 0.001
**Pulmonary tuberculosis**						
No	1.00					
Yes	3.51 (0.49;25-16)			0.211		
**Other factors**						
Multidrug-resistant tuberculosis	1.07 (0.72;1.57)			0.723		
HIV positive	0.97 (0.67;1.42)			0.912		
Diabetes	0.41 (0.20;0.84)			0.016		
Alcohol abuse	0.87 (0.62;1.21)			0.422		
Illicit drug use	2.24 (1.59;3.16)			< 0.001	1.62 (1.12;2.34)	0.009
Tobacco smoking	0.90 (0.64;1.26)			0.558		
**Type of admission**						
New case/transfer	0.75 (0.45;1.25)			0.281		
Post-dropout	2.55 (1.72;3.77)			< 0.001	2.04 (1.37;3.04)	< 0.001

a) HRc: Crude hazard ratio; b) HRa: Adjusted hazard ratio.

Having diabetes was a protective factor for self-discharge in the crude analysis (HR = 0.41; 95%CI 0.20;0.84), but in the adjusted regression, only use of illicit drugs (HR = 1.62; 95%CI 1.12;2.34) continued to have association with self-discharge. 

**Figure 2:**
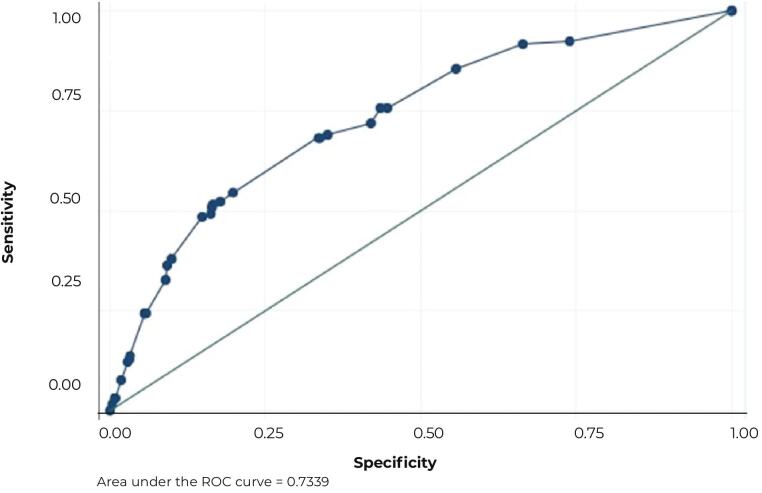
ROC curve showing distribution of sensitivity and specificity for different cut-off points for prediction of hospital self-discharge

Having dropped out of previous treatment was a predictive factor for self-discharge (HR = 2.04; 95%CI 1.37;3.04) when compared to those without previous treatment, new cases, or those who had been undergoing treatment in other health units and had been transferred to the hospital. The Harrell C statistic of the final adjusted model was 0.71 and the area under the ROC curve was 0.73 (Figure 2).

## DISCUSSION

The data showed that self-discharge was associated especially among people living on the streets or from shelters, users of illicit drugs, those under 42 years of age and with a history of having dropped out of previous treatment. These findings suggest that vulnerable living conditions and risk behaviors play a crucial role in self-discharge, highlighting the need for specific interventions for these populations. The female sex presented an increased risk for self-discharge, indicating that women may face specific challenges during hospitalization.

Given the retrospective nature of the study, data incompleteness of some variables may affect the validity of the results, even with imputation of missing data. Generalization of the findings is limited, since the study was conducted in a specific hospital in the state of Rio de Janeiro, and the results may not be applicable to other regions of Brazil or different hospital contexts. Despite these limitations, the study is original and consistent, as it has a significant sample size and follow-up time. Future studies should consider prospective, multicenter approaches to confirm these findings and provide a more comprehensive understanding of the factors that influence self-discharge among tuberculosis cases.

The average length of stay was 59 days and 23 days for cases of self-discharge. A study that analyzed hospitalizations for tuberculosis in a university hospital in São Paulo, showed average hospitalization of 29 days, with the longest hospitalization time being associated with drug use, smoking and side effects caused by medication.^16^ Another study, conducted in the state of São Paulo among patients hospitalized with tuberculosis, showed that the average length of stay was approximately 90 days, but, among patients with social vulnerability and alcohol abuse, average length of stay was 120 days. The long hospitalization time was justified by an understanding between hospitals, health centers and the Tuberculosis Program administration that adherence to treatment outside the hospital environment would be impaired in these groups, contributing to loss to follow-up if they were cared for as outpatients.^17^

The long length of stay among patients hospitalized for tuberculosis and the direct and indirect costs generated by the disease can be considered important factors for the unfavorable outcome of treatment.^18^ One of the social repercussions of tuberculosis is the difficulty in keeping a job or getting a job during treatment and in the event of complications during the course of the disease. Although treatment is free in Brazil, tuberculosis continues to result in catastrophic costs and reduced income for many families. One study showed that 61.5% of patients who dropped out of tuberculosis treatment were unemployed or working on the informal job market.^19^ These data reinforce the inability to work caused among economically active individuals, who are, in many situations, their family’s only income provider. The result is a worsening of the situation of poverty and social exclusion. 

Risk of self-discharge was 1.62 times greater among illicit drug users. Similarly, risk was two times greater among cases up to 42 years old. These data corroborate the results of a systematic review carried out with Brazilian studies, which suggest that being aged between 19 and 49 years, using alcohol and illicit drugs are risk factors associated with dropping out of tuberculosis treatment.^7^ In addition to the higher prevalence of young adults affected by tuberculosis, an aggravating factor is that there is increased propensity to use alcohol and drugs in this group.^20^

Patients who had already dropped out of previous tuberculosis treatment were twice as likely to self-discharge. The state of Rio de Janeiro stands out for its poor performance in tuberculosis treatment. In 2022, the treatment interruption rate among tuberculosis retreatment cases was 30.2%.^21^ Among the Brazilian states, Rio de Janeiro was in third place in terms of highest risk of becoming ill with tuberculosis (68.6 cases per 100,000 inhabitants) and had the highest tuberculosis mortality rate (4.4 deaths per 100,000 inhabitants).^22^ The low percentage of retreatment cases who underwent directly observed therapy in 2022 (35.9%), may also be a factor that explains performance below expected.^21^

In the sample studied, two thirds of the hospitalized individuals were male. The higher tuberculosis incidence rate and the higher rate of hospitalization due to the disease among male individuals have already been reported in the literature,^3,22^ as well as the higher risk of treatment interruption.^23^ Despite this, in the present study, hospitalized females showed 1.47 times more risk of self-discharge than males. The same was found in a study that analyzed type of discharge in five different hospitals for caring for patients with tuberculosis in São Paulo, where discharge on request was higher in the hospital that only admitted female patients.^24^ Half of Brazilian households are headed by women.^25^ Single-parent families with children and headed by females accounted for 15% of the household arrangements, while those with male heads of family accounted for 2%. The average per capita income of households headed by women was equivalent to 72% of households headed by men, with the lowest per capita income observed in single-parent households with children headed by females.^25^ Furthermore, the stigma related to tuberculosis also makes it possible to explain this result, as there is greater concealment and denial of the disease among women when compared to men, creating difficulties in seeking treatment.^26^

Risk of self-discharge was 2.5 times greater among people who lived on the streets or in shelters. Tuberculosis prevalence is up to 70 times higher in this population when compared to the general population. Despite this already known association, it is important to highlight that, in the period from 2018 to 2020, there was a 22% increase in incidence and a reduction in the cure of tuberculosis in this group, which went from 39.6% in 2014 to 33.9% in 2021. Between 2019 and 2021, 38% of new tuberculosis cases among street dwellers interrupted treatment. Additionally, during that period, an increase in the proportion of deaths among cases reported among street dwellers was seen.^22^ The risk of tuberculosis infection and therapeutic failure is more prevalent among this population, and can reach 57.3%.^27^ Therapeutic failure in these patients occurs mainly due to death (10.5%) or loss to follow-up (39%).^28^ There is also a strong association between abuse of illicit substances and people without a fixed place of residence.^29^

Faced with these challenges, the federal government established the Interministerial Committee for the Elimination of Tuberculosis and Other Socially Determined Diseases, as per Decree No. 11494 published in 2023, which ratified the multisectoral nature of addressing the disease. Articulation between political, administrative and operational sectors of the health system is essential in order to achieve comprehensive care for people who are most vulnerable to tuberculosis. This process must range from prevention to post-cure follow-up. It is essential to know and guarantee social rights for people undergoing treatment for tuberculosis, as well as for those in vulnerable situations.

The hospital environment can be even more challenging due to the need to adapt to previously non-existent confinement. The basic in-hospital support offered to these socially vulnerable individuals can facilitate adherence and success in their tuberculosis treatment. To reduce dropout rates and improve adherence to treatment, comprehensive care for patients is necessary, which encompasses aspects of physical health and psychosocial care. 

Adequate treatment involves continuous and long follow-up, which not only depends on the effectiveness of medication but also on adherence by the patient. Lack of access to health services, long treatment time, the stigma and costs of the disease, the social vulnerability of patients and the difficulty of reconciling work with treatment are just some of the aspects that make the situation of treatment interruption clear, both in the outpatient and the inpatient context. 

In conclusion, in the case of treatment that requires hospitalization for a prolonged period, hospital self-discharge mainly affects people facing vulnerable situations. It is essential that public policies be developed that guarantee adequate care, adjusted to social issues, aspects of physical health and psychosocial care that involve the fight against tuberculosis.
