# Supplementary material for: Predictive factors for hospital self-discharge in tuberculosis admissions in the state of Rio de Janeiro, from 2011-2018: a retrospective cohort study
Source: Epidemiol Serv Saude. 2024 Oct 14;33:e20231202. doi: 10.1590/S2237-96222024v33e20231202.en (PMC11473068; doi:10.1590/S2237-96222024v33e20231202.en)
Supplement: Translation

# Translation: Fatores preditivos para a evasão hospitalar nas internações por tuberculose no estado do Rio de Janeiro, de 2011 a 2018: estudo de coorte retrospectivo

**DOI:** 10.1590/S2237-96222024v33e20231202.pt

**Authors:** Marcela Bhering, Caroline Millon, Maria Eduarda Beltrão da Rosa Rinaldi, Hedi Marinho de Melo Guedes de Oliveira

**Affiliations:** 1 Fundação Oswaldo Cruz, Escola Nacional de Saúde Pública Sergio Arouca, Rio de Janeiro, RJ, Brasil; 2 Universidade do Grande Rio, Curso de Medicina, Rio de Janeiro, RJ, Brasil

**Keywords:** Tuberculose, Evasão do Paciente, Pacientes Internados, Fatores de Risco, Vulnerabilidade Social, Adesão ao Tratamento

## Abstract

**Objetivo:**

Analisar fatores associados à evasão hospitalar de pacientes com tuberculose no estado do Rio de Janeiro, de 2011 a 2018.

**Métodos:**

Trata-se de estudo de coorte retrospectivo em hospital de referência. Foram comparadas as características clínicas e epidemiológicas segundo desfecho da internação (evasão, alta ou óbito). *Hazard ratios* (HR) com intervalos de confiança de 95% (IC_95_%) da associação da evasão com as variáveis explicativas foram estimados por regressão de Cox.

**Resultados:**

Das 1.429 internações, 10,4% tiveram evasão como desfecho. Sexo feminino (HR = 1,47; IC_95_% 1,03;2,11), idade ≤ 42 anos (HR = 2,01; IC_95_% 1,38;2,93), uso de drogas (HR = 1,62; IC_95_% 1,12;2,34), internação após abandono de tratamento (HR = 2,04; IC_95_% 1,37;3,04) e pessoas vivendo em situação de rua (HR = 2,50; IC_95_% 1,69;3,69) foram associados à evasão.

**Conclusão:**

Pacientes com vulnerabilidade social necessitam de monitoramento mais cuidadoso durante a internação.

## Contribuições do estudo

**Principais resultados:**

População em situação de rua, uso de drogas ilícitas, sexo feminino e histórico de abandono de tratamento prévio mostraram associação com a evasão hospitalar em pacientes com tuberculose internados em hospital de referência no estado do Rio de Janeiro.

**Implicações para os serviços:**

Necessidade de suporte mais abrangente aos pacientes vulneráveis, além da promoção da adesão ao tratamento e o treinamento de profissionais de saúde para lidar com as complexas questões psicossociais relacionadas à tuberculose.

**Perspectivas:**

É crucial o desenvolvimento de políticas públicas que considerem fatores sociais na gestão da tuberculose, bem como a promoção da cooperação e abordagens multissetoriais para abordar tanto a tuberculose quanto as questões sociais subjacentes.

## INTRODUÇÃO

A tuberculose permanece um grave problema de saúde pública. A Organização Mundial da Saúde (OMS) estimou que, em 2022, 10,6 milhões de pessoas adoeceram em todo o mundo e cerca de 1,3 milhão de pessoas morreram com tuberculose, incluindo 187 mil entre pessoas HIV positivas.^1^ O Brasil é considerado um país prioritário no controle da tuberculose devido à alta carga da doença e à coinfecção com o HIV.^1^ Diante da necessidade de diminuir a carga dessa doença, a OMS tem destacado a importância do combate à tuberculose em populações vulneráveis e a necessidade de intervenções eficazes em nível nacional e mundial.^1^

No Brasil, o diagnóstico e o tratamento são oferecidos de forma gratuita e o Ministério da Saúde recomenda que os casos novos e de retratamento em uso de esquema básico, casos de recidiva após cura e de reingresso após interrupção terapêutica sejam tratados em regime ambulatorial na rede de atenção primária à saúde. Casos que necessitem de esquemas terapêuticos especiais, devido à toxicidade, intolerância ou contraindicações à adoção do esquema básico, e de avaliação de falência terapêutica, devem ser tratados nas unidades de referência secundárias, enquanto casos de resistência comprovada ou de falência terapêutica por resistência devem ser encaminhados para as unidades de atenção terciária.^2^

Apesar de não exercer papel central no tratamento da tuberculose, os hospitais ainda são uma importante porta de entrada para o diagnóstico da doença, especialmente nos médios e grandes centros urbanos.^2,3^ A proporção de casos de tuberculose diagnosticados no nível hospitalar é maior do que o esperado em várias regiões do país, seja em decorrência das barreiras de acesso à atenção primária a saúde, seja por atraso no diagnóstico.^4^ Estima-se que 30% dos casos de tuberculose sejam diagnosticados em hospitais, após se agravarem e necessitarem de internação, resultando em maiores despesas médicas e aumento das taxas de mortalidade.^4,5^

Sabe-se que a interrupção do tratamento é um enorme desafio para atingir as metas preconizadas pela OMS no plano de eliminação da tuberculose.^1,6^ De acordo com estudos realizados, os fatores associados à interrupção do tratamento de tuberculose são múltiplos e, de modo geral, estão associados ao paciente (coinfecção com HIV, uso de álcool, tabaco e drogas), a fatores socioeconômicos (baixa escolaridade, falta de moradia), à modalidade do tratamento empregado e à sua operacionalização nos serviços de saúde (falha no uso do tratamento observado diretamente).^7^

No Brasil, o Ministério da Saúde recomenda internação hospitalar para o tratamento da tuberculose nos casos em que há hemoptise, deterioração do estado geral do paciente, presença de comorbidades significativas, intolerância grave aos medicamentos, desenvolvimento de hepatite medicamentosa e situações de vulnerabilidade social.^8^ Em hospitais de longa permanência, os pacientes muitas vezes necessitam permanecer durante todo o período de tratamento e/ou de restabelecimento de sua saúde, devido às condições precárias de moradia, falta de suporte familiar ou outras dificuldades relacionadas ao contexto social.^8^ No contexto hospitalar, a evasão das internações por tuberculose, definida como a saída do paciente do ambiente hospitalar sem autorização médica e sem comunicação ao setor de internação,^9^ torna-se uma causa importante de interrupção do tratamento, contribuindo para desfechos desfavoráveis, como multirresistência aos fármacos e óbito.^4^

A evasão é um desafio para controle da doença e problema relevante na gestão das estratégias de saúde pública, uma vez que a interrupção do curso adequado do tratamento pode levar a uma piora do estado de saúde do paciente, aumento do risco de reinternações e até mesmo óbito. Além disso, pode resultar em maior transmissão da doença na comunidade, comprometer a eficácia dos medicamentos e aumentar o risco de resistência a eles. Apesar de haver na literatura estudos sobre abandono do tratamento da tuberculose,^7^ não há estudos que investiguem evasão dos pacientes hospitalizados por tuberculose. Diante disso, este artigo tem como objetivo identificar fatores associados à evasão hospitalar de pacientes com tuberculose. 

## MÉTODOS

Trata-se de um estudo de coorte retrospectivo realizado no Hospital Estadual Santa Maria, com dados de pacientes internados entre janeiro de 2011 e dezembro de 2018. 

O hospital possui 77 leitos destinados exclusivamente à internação de pacientes adultos com tuberculose, coinfecção com HIV, tuberculose multirresistente (TB-MDR), com média de 200 internações por ano. Por ser hospital de referência, os pacientes são encaminhados com o diagnóstico de tuberculose ativa. Das duas unidades hospitalares de referência para tuberculose no estado, é a única localizada no município do Rio de Janeiro, com média de 200 internações por ano no período do estudo. O estado do Rio de Janeiro (RJ) apresenta incidência alta de casos de tuberculose, com uma taxa de 70,7 casos por 100 mil habitantes.^10^

Os dados foram coletados diretamente dos prontuários por duas sanitaristas do Núcleo Hospitalar de Epidemiologia do hospital, responsáveis pela vigilância epidemiológica dos casos.

A população de referência foi constituída de pacientes adultos (≥ 18 anos de idade) internados no período do estudo. Foram incluídos todos os pacientes com diagnóstico confirmado de tuberculose, seja por detecção por teste direto (método de Ziehl-Neelsen), resultado positivo de cultura para *Mycobacterium tuberculosis*, seja pela presença de achados clínicos, epidemiológicos e radiográficos compatíveis com tuberculose, de acordo com a localização da doença.

Os desfechos foram classificados de acordo com o tipo de saída da internação, em conformidade com a Portaria nº 312, de 30 de abril de 2002:^9^ evasão – saída do paciente do hospital sem autorização médica e sem comunicação da saída ao setor em que o paciente estava internado; alta – ato médico que determina a finalização da internação hospitalar, podendo ser cura ou melhora; óbito – morte ocorrida após o paciente ter dado entrada no hospital, tendo a tuberculose como uma das causas. 

As seguintes variáveis explicativas foram selecionadas nos prontuários e incluídas no estudo, seguindo racional prévio:^11^

a) Sociodemográficas

- sexo (masculino; feminino);
- faixa etária (em anos: 18-30; 31-44; 45-64; ≥ 65);
- raça/cor da pele (branca, preta, parda, amarela, indígena, ignorado);
- escolaridade (em anos: nenhum; 1-3; 4-7; 8-11; ≥ 12);
- morador da capital (sim; não).

b) Clínicas

- forma clínica da tuberculose (pulmonar; extrapulmonar; ambas);
- tuberculose multirresistente (resistência a pelo menos rifampicina e isoniazida) (sim; não);
- tipo de entrada (caso novo; pós-abandono; transferência).

c) Comorbidades

- HIV (positivo, negativo, não realizado);
- diabetes (sim; não);

d) Fatores de risco

- dependência de álcool (sim; não);
- uso de drogas ilícitas (sim; não);
- tabagismo (sim; não);
- reingresso no tratamento após abandono (sim; não);
- paciente vivendo em situação de rua ou proveniente de abrigo público (sim; não).

Para a análise de regressão, foram utilizadas variáveis binárias para idade (≤ 42 anos) e escolaridade (< 8 ≥ 8 anos), definidas com base em estudos anteriores e testadas para o melhor ajuste do modelo.^12,13^ Para as variáveis que apresentavam incompletude de mais de 10% dos dados (escolaridade, morador da capital, forma da tuberculose), foi realizada imputação múltipla por regressão pelo método da média predita para prever dados faltantes.^14^

Dada a natureza das variáveis, frequências absolutas e relativas foram utilizadas para descrever as características dos pacientes e a ocorrência ou não do desfecho. O teste exato de Fisher foi utilizado para comparar as proporções entre o grupo que evadiu e os grupos que tiveram alta ou foram a óbito. Também foram calculadas as medianas e o intervalo interquartil (25-75) do tempo de permanência de acordo com o tipo de saída hospitalar.

A partir do mesmo conjunto de participantes, modelos de riscos proporcionais de Cox foram usados para estimar *hazard ratio* r (HR) entre o desfecho da evasão e as variáveis explicativas. O tempo-zero da coorte foi definido pela data de internação, e o tempo de seguimento correspondeu ao período desde a data da internação até o desfecho. Para os pacientes que não atingiram o desfecho de interesse (evasão), os dados foram censurados na última data conhecida de acompanhamento.

Foi aplicado o método de seleção proposital de Hosmer e Lemeshow, que é semelhante ao método *backward*.^15^ No modelo inicial, foram incluídas variáveis com o p-valor ≤ 0,20 na análise univariável, o que permite uma exploração inicial abrangente das associações com a variável dependente. Isso ajuda a identificar potenciais fatores de interesse, minimizando o risco de erro do tipo II e priorizando variáveis para análise adicional, juntamente com a teoria e o refinamento das variáveis, com apoio da literatura. Na etapa seguinte, as variáveis nas quais não se rejeitava a hipótese nula e que não provocavam uma alteração maior do que 20% nas estimativas dos coeficientes foram removidas do modelo, uma de cada vez. No modelo ajustado, foram incluídas variáveis associadas ao desfecho com significância ≤ 0,10. O modelo com melhor perfilamente aos dados foi escolhido por meio do teste da razão de verossimilhança. Os resíduos de Schoenfeld foram usados para testar o pressuposto da proporcionalidade e os resíduos de Cox-Snell foram usados para avaliar a qualidade geral do ajuste do modelo. Um nível de significância fixo em 5% foi tomado como ponto de corte para todos os testes estatísticos. Para avaliação da capacidade discriminativa do modelo, foi utilizada a estatística C de Harrell e a curva ROC *(receiver operating characteristic).* As análises estatísticas foram realizadas com o programa Stata versão 13.1.

O estudo foi aprovado pelo Comitê de Ética em Pesquisa da Escola Nacional de Saúde Pública Sergio Arouca/Fiocruz (Certificado de Apresentação de Apreciação Ética 44735121.5.0000.5240), com parecer nº 4.641.397, em 9 de abril de 2021.

## RESULTADOS

Entre 2011 e 2018, 1.512 pacientes foram internados no hospital. Foram excluídos 65 registros por falta de informação completa e 18 que não eram casos de tuberculose (mudança de diagnóstico), restando 1.429 registros. Do total dos 1.429 casos, 149 (10,4%) foram saídas por evasão, 288 (20,1%) foram óbito e 992 (69,5%) altas, sendo 145 (10,2%) por cura e 847 (59,3%) por melhora clínica. No geral, a mediana do tempo de internação foi de 59 dias (intervalo interquartil 19-121) e de 23 dias para evasão (Figura 1). 

**Figura 1:** Frequência de pacientes, mediana de tempo e intervalo interquartil (em dias), por desfecho, Rio de Janeiro, 2011 a 2018 (n= 1429)

A cidade do Rio de Janeiro foi responsável por 72,2% dos casos analisados, seguida pela região da Baixada Fluminense, com 226 (19,3%). Duque de Caxias e São João de Meriti foram os municípios da Baixada Fluminense que mais tiveram pacientes internados, 66 (5,6%) e 56 (4,7%), respectivamente.

A Tabela 1 descreve as características sociodemográficas e clínicas da população de estudo por desfecho (evasão, óbito ou alta). No geral, 1.063 (74%) pacientes eram do sexo masculino. Pacientes do sexo feminino estiveram proporcionalmente mais presentes entre os casos de evasão (30,9%), que nos grupos de alta (25%) e óbito (21,9%), mas sem diferença estatística significante (p = 0,115). Entre as internações analisadas, a mediana de idade foi de 42 anos (intervalo interquantil 32-54) nos pacientes em geral e de 35 anos (intervalo interquartil 30-43) entre os que evadiram. 

**Tabela 1:** Características sociodemográficas e clínicas dos participantes por tipo de saída hospitalar, Rio de Janeiro, 2011-2018 (n=1.429)

Características	Evasão (%)	Óbito (%)	Alta (%)	p-valor^a^
	**N = 149**	**N = 288**	**N = 992**	
**Sexo**				
Masculino	103 (69,1)	225 (78,1)	735 (75)	0,115
Feminino	46 (30,9)	63 (21,9)	257 (25)	
**Faixa etária** ^b^				
18-30	37 (25,5)	36 (12,7)	213 (21,9)	< 0,001
31-44	73 (50,3)	68 (24,0)	338 (34,8)	
45-64	34 (23,5)	136 (48,1)	372 (38,3)	
≥ 65	1 (0,7)	43 (15,2)	48 (5,0)	
**Raça/cor da pele** ^b^				
Branca	24 (18,2)	71 (26,1)	201 (21,9)	0,335
Parda	56 (42,4)	112 (41,2)	407 (44,4)	
Preta	52 (39,4)	89 (32,7)	308 (33,6)	
**Escolaridade (anos)** ^b^				
Nenhum	7 (12,7)	13 (9,7)	35 (8,6)	0,665
1 a 3	19 (34,6)	43 (32,1)	140 (34,2)	
4 a 7	15 (27,3)	48 (35,8)	120 (29,3)	
8 a 11	11 (20)	18 (13,4)	67 (16,4)	
≥ 12	3 (5,4)	12 (9,0)	35 (8,6)	
**Morador da capital** ^b^				
Sim	91 (79,8)	171 (68,7)	586 (73,6)	0,074
Não	23 (20,2)	78 (32,3)	210 (26,4)	
**População em situação de rua/abrigo**				
Sim	36 (24,2)	13 (4,5)	83 (8,4)	< 0,001
Não	113 (75,8)	275 (95,5)	909 (91,6)	
**Forma clínica da tuberculose** ^b^				
Pulmonar	113 (99,1)	224 (96,1)	707 (95,4)	0,581
Extrapulmonar	0 (0,0)	3 (1,3)	12 (1,6)	
Ambas	1 (0,9)	6 (2,6)	22 (3,0)	
**Outros fatores**				
TB-MDR	34 (22,8)	52 (18,1)	193 (19,5)	0,481
HIV positivo	37 (24,8)	79 (27,4)	241 (24,3)	0,549
Diabetes	8 (5,3)	31 (10,7)	123 (12,4)	0,03
Abuso de álcool	86 (57,7)	149 (51,7)	557 (56,2)	0,352
Uso de drogas ilícitas	97 (65,1)	74 (25,7)	437 (44,1)	< 0,001
Tabagismo	90 (60,4)	142 (49,3)	583 (58,8)	0,012
**Tipo de entrada** ^b^				
Caso novo	18 (13,6)	55 (21,2)	196 (21,4)	0,001
Pós-abandono	34 (25,8)	25 (9,7)	106 (11,2)	
Transferência	80 (60,6)	179 (69,1)	614 (67,0)	

a) Teste exato de Fisher; b) Variável com menos informações, devido à incompletude dos dados no prontuário.

Entre os casos com escolaridade conhecida (n = 598), 440 (73,6%) tinham até sete anos de estudo. Entre os casos de evasão, houve maior proporção de casos sem nenhuma escolaridade (12,7%), que entre os casos de óbito (9,7%) e alta (8,6%), sem diferença significante entre os grupos (p = 0,665).

Entre os casos de evasão, 24,2% foram de pessoas vivendo em situação de rua ou oriundas de abrigos; alta e óbito nessa população foram menores, 8,4% e 4,5%, respectivamente (p < 0,001). Pacientes que utilizavam drogas ilícitas foram proporcionalmente maiores entre casos de evasão (65,4%), do que entre alta (44,1%) e óbito (25,7%; p < 0,001). O mesmo ocorreu entre pacientes tabagistas, com maior proporção entre casos de evasão (60,4%), do que de alta (58,8%) e óbito (49,3%; p = 0,012). 

Pacientes TB-MDR tiveram maior percentual de casos no desfecho evasão (22,8%). Já no desfecho óbito, houve maior proporção de casos HIV positivos (27,4%), enquanto nas altas houve mais casos de diabetes (12,4%). A frequência de pacientes que haviam abandonado o tratamento anterior foi maior entre casos de evasão (25,8%), quando comparados aos casos de óbito (9,7%) e alta (11,2%) (p < 0,001). 

Após ajustes, indivíduos do sexo feminino apresentaram maior risco de evasão (HR = 1,47; IC_95_% 1,03;2,11) quando comparados aos do sexo masculino (Tabela 2). Pacientes com idade ≤ 42 anos apresentaram duas vezes mais risco de evasão que pacientes mais velhos (HR = 2,01; IC_95_% 1,38;2,93). Pessoas vivendo em situação de rua ou em abrigos apresentaram mais risco de evasão hospitalar (HR = 2,50; IC_95_% 1,69;3,69) que pacientes com residência conhecida. 

**Tabela 2:** Fatores preditivos para evasão entre pacientes internados em hospital de referência para tuberculose, Rio de Janeiro, 2011 a 2018 (n=1.429)

Variáveis	Medida de associação bruta (IC_95_ **%**) HRb^a^ IC_95_ **%**	p-valor	Medida de associação ajustada (IC_95_ **%**) HRa^b^ IC_95_ **%**	p-valor
**Sexo**				
Masculino	1,0 0		1	
Feminino	1,54 (1,08;2,19)	0,016	1,47 (1,03;2,11)	0,033
**Idade ≤ 42 anos**				
Não	1		1	
Sim	2,54 (1,78;3,62)	< 0,001	2,01 (1,38;2,93)	< 0,001
**Raça/cor da pele**				
Branca	1			
Parda/preta	1,22 (0,77;1,92)	0,379		
**Escolaridade (anos)**				
< 8	1			
≥ 8	0,91 (0,49;1,68)	0,779		
**Morador da capital**				
Não	1			
Sim	1,39 (0,88;2,21)	0,152		
**População em situação de rua ou abrigo**				
Não	1		1	
Sim	2,99 (2,05;4,36)	< 0,001	2,50 (1,69;3,69)	< 0,001
**Tuberculose pulmonar**				
Não	1			
Sim	3,51 (0,49;25-16)	0,211		
**Outros fatores**				
Tuberculose multirresistente	1,07 (0,72;1,57)	0,723		
HIV positivo	0,97 (0,67;1,42)	0,912		
Diabetes	0,41 (0,20;0,84)	0,016		
Abuso de álcool	0,87 (0,62;1,21)	0,422		
Uso de drogas ilícitas	2,24 (1,59;3,16)	< 0,001	1,62 (1,12;2,34)	0,009
Tabagismo	0,90 (0,64;1,26)	0,558		
**Tipo de entrada**				
Caso novo/transferência	0,75 (0,45;1,25)	0,281		
Pós-abandono	2,55 (1,72;3,77)	< 0,001	2,04 (1,37;3,04)	< 0,001

Ter diabetes foi fator protetor para evasão na análise bruta (HR = 0,41; IC_95_% 0,20;0,84), mas, na regressão ajustada, apenas o uso de drogas ilícitas (HR = 1,62; IC_95_% 1,12;2,34) manteve associação com a evasão. 

Abandono de tratamento anterior foi fator preditivo para evasão (HR = 2,04; IC_95_% 1,37;3,04), quando comparado aos sem tratamento anterior, casos novos, ou com os que estavam realizando tratamento em outras unidades de saúde e foram transferidos para o hospital. A estatística C de Harrell do modelo ajustado final foi de 0,71 e a área sob a curva ROC foi de 0,73 (Figura 2).

**Figura 2:** Curva ROC mostrando a distribuição da sensibilidade e especificidade para diferentes pontos de corte na predição da evasão hospitalar

## DISCUSSÃO

Os dados mostraram que a evasão esteve associada especialmente a pessoas vivendo em situação de rua ou oriundas de abrigos, usuários de drogas ilícitas, com menos de 42 anos e com histórico de abandono de tratamento anterior. Esses achados sugerem que condições de vida vulneráveis e comportamentos de risco desempenham um papel crucial na evasão hospitalar, destacando a necessidade de intervenções específicas para essas populações. O sexo feminino apresentou risco aumentado para evasão, indicando que as mulheres podem enfrentar desafios específicos durante a internação.

Dada a natureza retrospectiva do estudo, a incompletude dos dados em algumas variáveis pode afetar a validade dos resultados, mesmo com a imputação de dados ausentes. A generalização dos achados é limitada, pois o estudo foi conduzido em um hospital específico no estado do Rio de Janeiro, e os resultados podem não ser aplicáveis a outras regiões do Brasil ou a diferentes contextos hospitalares. Apesar dessas limitações, o estudo é original e consistente, por ter uma amostra e tempo de seguimento expressivos. Estudos futuros devem considerar abordagens prospectivas e multicêntricas para confirmar esses achados e fornecer uma compreensão mais abrangente dos fatores que influenciam a evasão hospitalar em pacientes com tuberculose.

O tempo médio de internação foi de 59 dias e de 23 dias para casos de evasão. Estudo que analisou as hospitalizações por tuberculose, em um hospital universitário em São Paulo, mostrou uma média de 29 dias de internação, sendo o maior tempo de internação associado ao uso de drogas, tabagismo e efeitos colaterais dos fármacos.^16^ Outro estudo, conduzido no estado de São Paulo entre pacientes internados com tuberculose, evidenciou que o tempo médio de internação era de aproximadamente 90 dias, mas, entre pacientes com vulnerabilidade social e abuso de álcool, esse tempo foi de 120 dias. O longo tempo de internação foi justificado por um entendimento entre os hospitais, as unidades de saúde e a coordenação do programa de tuberculose de que a adesão terapêutica, fora do ambiente hospitalar, seria prejudicada nesses grupos, contribuindo para a perda de seguimento em regime ambulatorial.^17^

O longo período de internação de pacientes hospitalizados por tuberculose e os custos diretos e indiretos gerados pela doença podem ser considerados importantes fatores para o desfecho desfavorável do tratamento.^18^ Uma das repercussões sociais da tuberculose é a dificuldade de a pessoa manter-se trabalhando ou conseguir emprego durante o tratamento e em caso de agravos no curso da doença. Embora o tratamento seja gratuito no Brasil, a tuberculose continua resultando em custos elevados e diminuição da renda para muitas famílias. Estudo mostrou que 61,5% dos pacientes que abandonam o tratamento de tuberculose estavam desempregados ou inseridos no mercado de trabalho informal.^19^ Esses dados reforçam a incapacidade ao trabalho provocada em indivíduos economicamente ativos, os quais são, em muitas situações, os únicos provedores de renda das famílias. O resultado é o agravamento da situação de pobreza e exclusão social. 

Usuários de drogas ilícitas apresentaram 1,62 vezes mais risco de evasão hospitalar, assim como pacientes com até 42 anos, que tiveram duas vezes mais evasão. Esses dados corroboram os resultados de revisão sistemática realizada com estudos brasileiros, que sugerem que idade entre 19 e 49 anos, uso de álcool e drogas ilícitas são fatores de risco associados ao abandono do tratamento da tuberculose.^7^ Além da prevalência mais elevada de adultos jovens afetados pela tuberculose, como agravante, há a propensão aumentada ao uso de álcool e drogas nesse grupo.^20^

Pacientes que já haviam abandonado tratamento anterior para tuberculose tiveram duas vezes mais evasão. O estado do Rio de Janeiro se destaca pelo seu fraco desempenho no tratamento da tuberculose. Em 2022, a taxa de interrupção do tratamento, entre casos de retratamento da tuberculose, foi de 30,2%.^21^ O estado foi o terceiro do país com maior risco de adoecimento por tuberculose (68,6 casos por 100 mil habitantes) e teve a maior taxa de mortalidade pela doença (4,4 óbitos por 100 mil habitantes).^22^ O baixo percentual de casos de retratamento que fizeram tratamento diretamente observado em 2022 (35,9%) também pode ser um fator que explique o desempenho aquém do esperado.^21^

Na amostra estudada, dois terços dos indivíduos internados eram do sexo masculino. A maior taxa de incidência de tuberculose e a maior taxa de hospitalização pela doença entre indivíduos do sexo masculino já foram reportadas na literatura,^3,22^ assim como o maior risco de interrupção do tratamento.^23^ Apesar disso, no presente estudo, as internações de pessoas do sexo feminino apresentaram 1,47 vez mais risco de evasão do que as do sexo masculino. O mesmo foi observado em estudo que analisou o tipo de alta em cinco diferentes hospitais para internação de pacientes com tuberculose em São Paulo, onde a alta a pedido foi maior na unidade que internava apenas pacientes do sexo feminino.^24^ Metade dos lares brasileiros são liderados por mulheres.^25^ Famílias monoparentais com filhos e chefia feminina representaram 15% dos arranjos, enquanto aquelas com chefia masculina representavam 2%. A renda *per capita* média dos domicílios chefiados por mulheres foi equivalente a 72% daquela dos domicílios liderados por homens, sendo a menor renda *per capita* observada nos domicílios monoparentais com chefes mulheres e com filhos.^25^ Além disso, o estigma relacionado à tuberculose também torna possível a explicação para esse resultado, visto que há maior ocultação e negação da doença entre mulheres quando comparadas aos homens, gerando dificuldades na busca pelo tratamento.^26^

A população vivendo em situação de rua ou em abrigos teve um risco 2,5 vezes maior de evasão. A prevalência de tuberculose é até 70 vezes superior nessa população, quando comparada à população geral. Apesar dessa associação já conhecida, é importante ressaltar que, no período de 2018 a 2020, houve aumento de 22% na incidência e redução da cura de tuberculose nesse grupo, que passou de 39,6%, em 2014, para 33,9% em 2021. Entre 2019 e 2021, 38% dos casos novos de tuberculose entre pessoa em situação de rua tiveram como desfecho a interrupção do tratamento. Adicionalmente, observou-se nesse período o aumento da proporção de óbitos entre os casos notificados entre pessoa em situação de rua.^22^ O risco de infecção por tuberculose e de falência terapêutica é mais prevalente entre essa população, podendo chegar a 57,3%.^27^ O insucesso terapêutico nesses pacientes ocorre sobretudo por morte (10,5%) ou perda de seguimento (39%).^28^ Existe ainda uma forte associação entre o abuso de substâncias ilícitas e pessoas sem residência fixa.^29^

Diante desses desafios, o governo federal instituiu o Comitê Interministerial para a Eliminação da Tuberculose e de Outras Doenças Determinadas Socialmente, Decreto nº 11.494, em 2023, que ratificou o caráter multissetorial do enfrentamento à doença. É essencial a articulação entre setores políticos, administrativos e operacionais do sistema de saúde com o objetivo do cuidado integral às pessoas que apresentam maior vulnerabilidade à tuberculose. Esse processo deve englobar desde a prevenção até o seguimento pós-cura. É essencial o conhecimento e a garantia dos direitos sociais para as pessoas que estão em tratamento para a tuberculose, como também para aquelas que se encontram em situações vulneráveis.

O ambiente hospitalar pode ser ainda mais desafiante, pela necessidade de adaptação ao confinamento previamente inexistente. O suporte básico intra-hospitalar ofertado a esses indivíduos socialmente vulneráveis pode facilitar a adesão e o sucesso do tratamento da tuberculose nessa população. Para diminuir a evasão e melhorar a adesão ao tratamento, é necessário um cuidado integral aos pacientes, que engloba aspectos da saúde física e a atenção psicossocial. 

O tratamento adequado envolve um seguimento contínuo e longo, que não depende apenas da eficácia de um medicamento, mas também da adesão pelo próprio paciente. A carência de acesso aos serviços de saúde, o longo tempo de tratamento, o estigma e os custos da doença, a vulnerabilidade social dos pacientes e a dificuldade de conciliar trabalho e tratamento são apenas alguns dos aspectos que ajudam a elucidar o quadro de interrupção do tratamento, tanto no contexto ambulatorial quanto no hospitalar. 

Conclui-se que, em um tratamento que exige internação por um período prolongado, a evasão hospitalar atinge principalmente pessoas com alguma situação de vulnerabilidade. Torna-se essencial desenvolver políticas públicas que garantam atendimento adequado, ajustado às questões sociais, aos aspectos da saúde física e à atenção psicossocial que circundam o enfrentamento da tuberculose.
